# Evidence of the Most Stretchable Egg Sac Silk Stalk, of the European Spider of the Year *Meta menardi*

**DOI:** 10.1371/journal.pone.0030500

**Published:** 2012-02-08

**Authors:** Emiliano Lepore, Andrea Marchioro, Marco Isaia, Markus J. Buehler, Nicola M. Pugno

**Affiliations:** 1 Laboratory of Bio-inspired Nanomechanics “Giuseppe Maria Pugno”, Department of Structural Engineering, Politecnico di Torino, Torino, Italy; 2 Laboratory of Ecology and Terrestrial Ecosystems, Department of Human and Animal Biology, University of Torino, Torino, Italy; 3 Laboratory for Atomistic and Molecular Mechanics, Department of Civil and Environmental Engineering, Center for Materials Science and Engineering, and Center for Computational Engineering, Massachusetts Institute of Technology, Cambridge, Massachusetts, United States of America; 4 National Institute of Nuclear Physics, National Laboratories of Frascati, Frascati, Italy; 5 National Institute of Metrological Research, Torino, Italy; University of Western Ontario, Canada

## Abstract

Spider silks display generally strong mechanical properties, even if differences between species and within the same species can be observed. While many different types of silks have been tested, the mechanical properties of stalks of silk taken from the egg sac of the cave spider *Meta menardi* have not yet been analyzed. *Meta menardi* has recently been chosen as the “European spider of the year 2012”, from the European Society of Arachnology. Here we report a study where silk stalks were collected directly from several caves in the north-west of Italy. Field emission scanning electron microscope (FESEM) images showed that stalks are made up of a large number of threads, each of them with diameter of 6.03±0.58 µm. The stalks were strained at the constant rate of 2 mm/min, using a tensile testing machine. The observed maximum stress, strain and toughness modulus, defined as the area under the stress-strain curve, are 0.64 GPa, 751% and 130.7 MJ/m^3^, respectively. To the best of our knowledge, such an observed huge elongation has never been reported for egg sac silk stalks and suggests a huge unrolling microscopic mechanism of the macroscopic stalk that, as a continuation of the protective egg sac, is expected to be composed by fibres very densely and randomly packed. The Weibull statistics was used to analyze the results from mechanical testing, and an average value of Weibull modulus (*m*) is deduced to be in the range of 1.5–1.8 with a Weibull scale parameter (*σ*_0_) in the range of 0.33–0.41 GPa, showing a high coefficient of correlation (R^2^ = 0.97).

## Introduction

Spider silks often display strong mechanical properties [1] and have been studied extensively during the last five decades. In particular, dragline silk is noted for its unique strength and toughness. Because of the complex structure of spider silk, large scale synthetic production still remains a challenge and can only be achieved through a controlled self-assembly of the macromolecular components with nanoscale precision [2].

Individual spiders spin ‘toolkits’ of seven to eight different types of silks, each of which comes from its own discrete gland(s) and spigot(s) [3]. Each type of spider silk has a unique chemical composition, molecular structure and material properties [4]. Orbwebs, for example, are composite structures built from multiple types of silks, each with its own unique molecular structure and mechanical function [4].

The best studied type of silk is the dragline silk, which is produced in the major ampullate gland. As the name itself suggests, dragline silk is used as a lifeline by most spiders moving through the environment and forms the backbone of most webs [4]. Minor ampullate glands produce threads that are sometimes added to major ampullate draglines or temporary spirals of the orbweb acting like a scaffolding for the construction of the web. Aciniform glands produce the silk used for prey wrapping and egg case construction and its fiber are more stretchable and tougher than dragline silk [5]. Flagelliform glands are unique to araneoid-orbweaving spiders and are used in the production of the catching spiral silk. In some derived taxa (like cobweb spinning theridiids) this type of silk is used to wrap preys [6]. Aggregate glands produce the glue coating on viscid capture threads and are unique to araneoid spiders, whilst piriform glands is used to cement threads to the substrate as well as to form silk junctions by forming attachment disks [4].

In line with state-of-the-art knowledge, it is widely accepted that a major role in the production of silk for egg sacs is played by the tubuliform (or cylindrical) glands [7]–[10], and it is likely that some spiders produce egg sac silk exclusively in these glands. Tubuliform silk is produced solely by adult orbweaving females. Egg sacs themselves are complex, layered structures containing fibres from several different glands [11]–[13]. This complexity creates confusion about how tubuliform silk is utilized. However, the morphology of the silk is quite distinctive because the glands produce large fibers with an irregular surface that is unlike any other silk. Moreover, the left and right fibers are coated with a gluey secretion that causes them to adhere together [11]. The mechanical behaviour of the silk is quite distinct in displaying a very prominent yield followed by a long low modulus extension [3], [4], [14].

**Figure 1:**
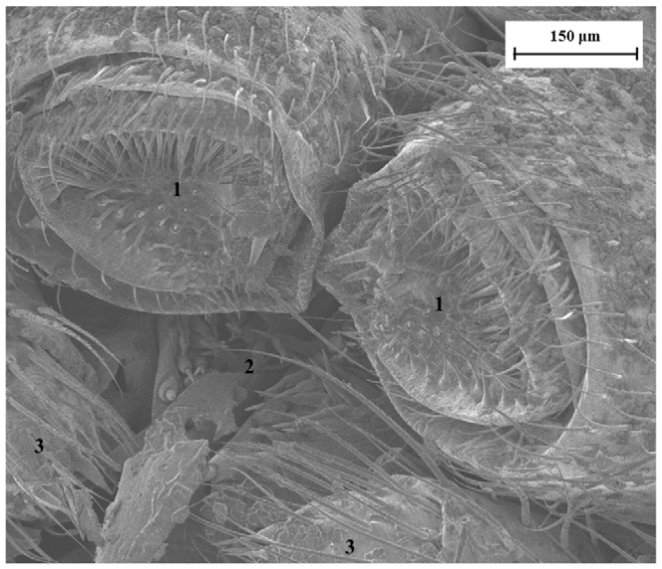
FESEM image of the spinnerets of *Meta menardi* (1. Anterior lateral; 2. Posterior median; 3. Posterior lateral).

In orbweb spiders, the spinnerets are three paired appendage-like organs on the abdomen, each of which contains dozens to hundreds of spigots connected to their own internal silk-producing glands (**Figure 1**) [15]. A single spider is therefore capable of producing multiple silk threads of many kinds, and the arrangement of spigots on the spinnerets appears to relate functionally to how different silks are used together [6]. Dragline silk, flagelliform silk, aggregate silk and aciniform silk have been extensively characterized in *Argiope trifasciata* (Forsskål) [16]–[21], *Araneus diadematus* (Linnaeus) [22]–[31], *Argiope argentata* (Fabricius) [3], [32], *Argiope bruennichi* (Scopoli) [33], *Araneus gemmoides* Chamberlin & Ivie [32], [34], *Larinioides ( = Araneus) sericatus* Clerck [35], *Nephila edulis* (Labillardière) [23], [25], *Nephila clavipes* (Linnaeus) [32], [34], [36]–[38], *Nephila pilipes* Fabricius [36], *Nephila madagascariensis* ( = *N. inaurata madagascariensis*) (Vinson) [29], *Lactrodectus hesperus* Chamberlin & Ivie [32], [39], *Leucauge venusta* Walckenaer [32], *Plectreurys tristis* Simon [32], *Kukulcania hibernalis* Hentz [32] and *Salticus scenicus* (Clerck) [30]. These studies have shown that the various types of silks, produced by different glands, have very different mechanical properties [22], [24], [34], giving the thread different characteristics, depending on their respective function [8], that may vary according to different species. Variability in the mechanical properties of spider silk is very important. Spider silk is in fact central to many aspects of spider biology and ecology, from communication to prey capture. Spiders are the only animals which use silk in almost every part of their daily lives. Because silk is so important to spiders, it has presumably been subjected to strong selective pressures during the 400 million years of spider evolution and can be regarded as one of the key to spider's evolutionary success [40], [41].

It has been demonstrated that silk properties (in terms of different reeling methods [32], [42], environmental conditions [8], [20], types of silk [e.g. dragline, viscid or egg sac silk] [22], [24], [34]) are species-specific and lead to silk-based peptide fibrils or protein aggregates with different structural and mechanical properties. For example, different reeling speeds cause a variation in the diameter of the dragline thread [25] and so depending on the thickness of the thread the stress-strain curve varies. Spider dragline silk was tested in a wet environment to show that moisture induces supercontraction in the threads for levels higher than 70–75% of relative humidity, proving that when a thread is exposed to moisture, stresses quickly build up and tighten the thread [20]. By varying the conditions under which the spiders were kept (different reeling speeds, starvation periods), the species or the spiders inside the same species, it has been seen that dragline silk has different mechanical properties and varies on an interspecific, intraspecific and intra-individual level [23].

All silks are proteinaceous and belong to the general class of hierarchical protein materials. Each thread of spider silk is a composite of semi-amorphous α-chains and β-pleated nanocrystals [43]. In the orb web spider *Araneus diadematus* (the common European garden spider), the β-sheets are made up of a series of highly conserved poly-Ala repeats and are stacked up, thereby forming the protein crystals; these are embedded in a matrix of loosely arranged glycine-rich amino acids [26]. These protein crystals are held together by hydrogen bonds, one of the weakest chemical bonds, and have an important role in defining the mechanical properties of silk. When an external force is applied, the loose amino acids stretch and from a disordered position are straightened, whilst the β-sheets are subject to tensile force [44]. The β-sheet rich crystalline units are responsible for the toughness of the silk thread while the remaining, apparently amorphous regions, have a rubber like behavior [45]. One study used a simple coarse-grained model to simulate the mechanical deformation of silk in which the silk constitutive unit was a combination of two domains representing the α-chains and β-pleated sheets [46]. The stress-strain curve of their simulation had a similar shape to that of silk.

The studies on dragline silk have given us the opportunity to find a natural fiber with strong tensile properties in terms of large deformation [3], [8], [17]–[19], [21]–[30], [32]–[37], [39], [47]. A recent study has discovered a dragline silk which is twice as tough as any other previously described silk: this silk belongs to *Caerostris darwini* Kuntner & Agnarsson, which is a spider which constructs its orb web suspended above streams, rivers and lakes [47]. To be able to thoroughly understand all the various properties of spider silk we must be able to characterize all the different kind of silk.

The stress-strain behavior of the egg sac silk of *Araneus diadematus* [24] presents a logarithmic behavior, which is completely different to the behavior of dragline and viscid silk. The same can be said about the egg sac silk of *Argiope bruennichi* [33]. The stress-strain curves of the egg sac silk start with a small elastic region and then present an extremely flat plastic-hardening region [24]. The strain to break is roughly the same as that of the dragline, but the tensile strength is about from 3 to 4 times lower. The egg case silk has an initial modulus, which is a measure of the stiffness of the fiber, significantly higher than that of the dragline thread. These differences are partly due to the different amino acid compositions in the silks. To our knowledge, few studies have been conducted on stalks of egg sac silk. In general, each egg sac consists of two major parts that can be distinguished by the naked eye, namely an egg sac case and a stalk. The egg sac case houses eggs, while the stalk attaches the cocoon to the substrate [12]. In the literature, the strain of spider egg sac silk is in the range from 19% for *Araneus gemmoides* [34] to 29% for *Argiope argentata* [3], showing an average value of ∼26%; while the average stress is of 1.1 GPa with a minimum value of 0.3 GPa for *Araneus diadematus* [24] and the maximum stress of 2.3 GPa for *Araneus gemmoides* [34].

One study took bundles of 100 dragline and minor ampullate silk threads respectively and pulled them at constant speeds [34]. They observed that physical interactions between the fibers influenced the elongation data and so increased the stretching capabilities of the bundle, compared to that of the single fiber. They saw that *Nephila clavipes* dragline silk had almost double the final stress value compared to the same silk of *Araneus gemmoides*, whilst the minor ampullate silk had roughly the same final stress value [34].

**Figure 2:**
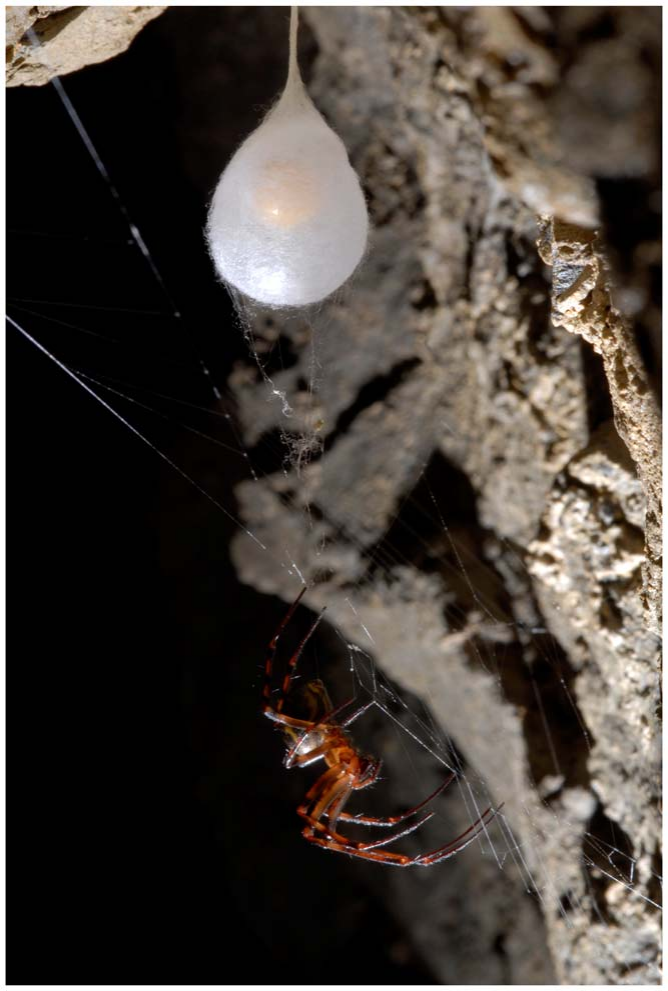
Egg sac of the spider *Meta menardi.* Photo by Francesco Tomasinelli (2009).

The cave spider *Meta menardi* (Latreille) is generally found in dark and humid places like caves and mines, throughout the northern hemisphere; and from northern Europe to Korea and northern Africa [16]. The cave spider *Meta menardi* has recently been chosen as the “European spider of the year 2012” from the European Society of Arachnology. Since no engineering studies of the egg sac of the cave spider *Meta menardi* yet exist and just few ones have been focused on egg sacs, we decided to conduct tensile tests on stalks of egg sac silk. We tested the stalk which connect the egg sacs of *Meta menardi* to the ceiling of the caves (the arrow, in **Figure 2**, indicates such sample). In total 15 stalks were found and were pulled until they broke. Samples were viewed under FESEM to analyze the fracture surfaces and measure the diameter of the stalk. To be able to see how the threads were stacked in each stalk, a Focused Ion Beam (FIB) was used to cut the stalk. Using the FESEM micrographies of the cross-section of the FIB-cut stalk and the processing software ImageJ 1.41o, we were able to measure the real diameter and the exact number of single threads in each stalk, improving the accurateness [19]. Thus, the stress-strain curves and the Weibull shape and scale parameters of the stalk of the egg sac silk of *Meta menardi* are here determined.

## Materials and Methods

Note that: no specific permits were required for the described field studies, the location is not privately-owned, the field studies did not involve endangered or protected species.

### Tensile testing

We identified different caves in Piedmont (a north-western region of Italy) to search for *Meta menardi* egg sacs. The egg sacs are generally spun at the end of summer and hatch in late winter. Fifteen stalks of the egg sacs were taken from the caves in which they were found (**Table 1**). Since the egg sacs were collected in their natural habitat, the measured mechanical stress-strain behavior of the silk would probably better represent the real characteristics than that produced by lab-reared spiders.

**Table 1:** List of the caves visited for the collection of the samples with collection date and number of samples.

Cave name	Speleological cadastre number	Municipality	Province	Date	Number of samples
Grotta del Bandito	1002 Pi/CN	Roaschia	Cuneo	02/2011	3
Grotta inferiore del Pugnetto or Tana del lupo	1502 Pi/TO	Mezzenile	Torino	02/2011	4
Grotta di Chiabrano or Tuna del Diau	1621 Pi/TO	Perrero	Torino	02/2011	8

We collected fifteen stalks of the egg sacs in three different caves: four in Grotta Inferiore del Pugnetto, three in Grotta del Bandito and eight in Grotta di Chiabrano. The spiders of this species are generally found in dark areas close to cave opening, where temperature and humidity are still influenced by the external conditions. The egg sacs hung from the ceiling of the cave with a bundle of threads (stalk) and are generally found in ventilated areas. The surveys were done on three separate days. When we found the egg sacs, we carefully took the stalks of the egg sacs from the ceiling of the caves and glued only the stalk ends to 30×50 mm^2^ cardboard holders, which had a ∼20×20 mm^2^ hole in their center so that the stalk could be suspended to enable the whole to be transported maintaining the original tension of the stalkand mounted on the testing machine without being damaged. All tests were done in the Laboratory of Bio-inspired Nanomechanics “Giuseppe Maria Pugno” (Politecnico di Torino, Italy) with an air temperature of 22±1°C and 31±2% relative humidity.

Tensile tests were conducted on thirteen of the fifteen specimens, the remaining two specimens were representatives of the tested samples and examined under the FESEM and FIB. The tensile tests were conducted using a testing machine (Insight 1 kN, MTS, Minnesota, USA), equipped with a 10 N cell load with pneumatic clamps (closure pressure of 275.6 kPa). The cardboard holders were placed between the clamps with an additional double-sided tape defining an initial length *l*_0_ in the range from 18 to 19 mm. Once the holders were in place, the clamps were brought to zero tension and then the sides of the holders were cut, leaving the stalk loose between the clamps. The specimens were pulled until they completely broke at a constant rate of 2 mm/min, coherently with the parameter setting of previous studies [20], [23], [24], [25], [29], [33], [34], [48].

The computer program TestWorks 4 (MTS, Minnesota, USA) recorded the experimental data of the applied tensile force and then the stress-strain curves were computed using the estimation of the real diameter and of the exact number *n* of single threads at the cross-section of each stalk. Stress *σ*, strain *ε* and modulus *E*, in order, were calculated using the following equations (1, 2, 3):
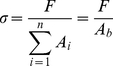
(1)

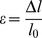
(2)

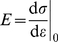
(3)where *F* is the force measured by the testing machine, *A_b_* is the initial cross-sectional area of the stalk (given as the initial cross-sectional area *A_i_* of a single thread multiplied by the number *n* of threads of the stalk), *l*_0_ is the initial length of the stalk and Δ*l* is the change in stalk length during test. The area under the stress-strain curve gives the energy required to break the material, and this variable can be used to quantify toughness. The spider silk dissipates energy in the volume, thus the classical fracture toughness cannot be defined, suggesting intrinsic huge toughening mechanisms.

The stress results of the tensile tests are then treated with the Weibull statistics, which defines the probability of failure *P* for a stalk as:
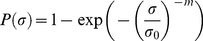
(4)where *σ* is the applied stress, *m* is the Weibull's shape parameter, or Weibull modulus, and *σ*_0_ is the Weibull's scale parameter. The cumulative probability *P*_i_(*σ*_i_) can be obtained experimentally as: 
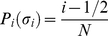
(5)where *N* is the total number of measured fracture stresses *σ*_i_, ranked in ascending order.

### FESEM and FIB Characterization

Each stalk was cut by FIB (FEI Quanta 3D FEG, at 5 kV). The real diameter and the exact number of single threads in each stalk was determined using the FESEM (FEI-Inspect™ F50, at 1–2 kV) micrographies of the cross-section of the FIB-cut stalk and the processing software ImageJ 1.41o.

## Results

**Figure 3:**
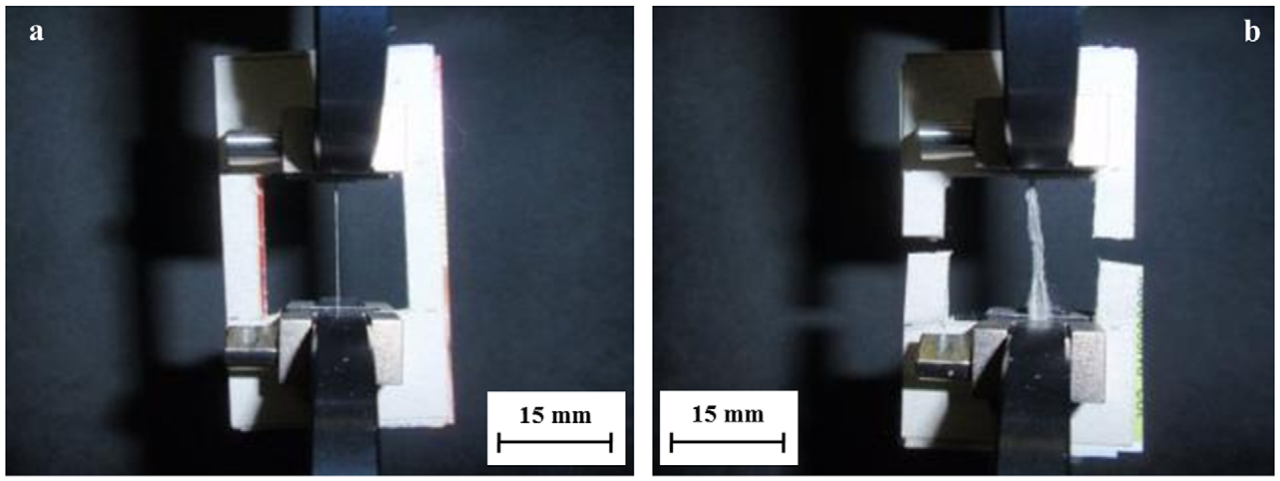
Distinction of the stalk types: cable-like (Group A) (a) and ropey-like (Group B) (b).

We performed tensile tests of the egg sac silk stalks of *Meta menardi*. The 13 stalks that we found were divided into two groups depending on the type of stalk. We were able to macroscopically distinguish two types of stalk “cable” type (group A) and “ropey” type (group B). The “cable” like stalk was made up of a series of threads tightly packed together forming a very compact structure (**Figure 3 a**), meanwhile in the “ropey” stalk the threads were not very compacted (**Figure 3 b**). Group A and B had 4 and 6 stalks, respectively. The remaining stalks did not give us concrete information in terms of tensile strength and were discarded. The tensile tests performed gave very different values in terms of stress, strain and modulus. This motivated us to interpret the results with Weibull statistics.

**Figure 4:**
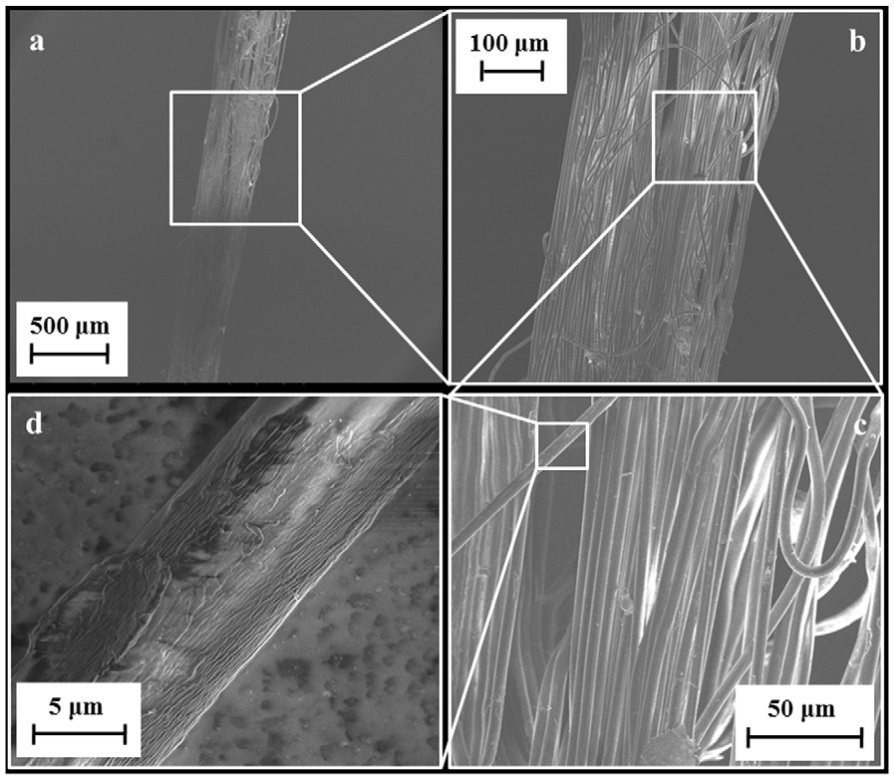
FESEM characterization of the silk stalk at different magnifications.

The FESEM images showed that the threads that made up the stalks all had similar diameters and all are clearly parallel-oriented (**Figure 4 a, b, c**). Thus, we were able to assess that each stalk was made up of a certain number of parallel threads, which are all with about the same diameter. In addition, their ends are clamped between the pneumatic clamps with an additional double-sided tape at the closure pressure of 275.6 kPa, which is a high pressure if compared to the testing forces. As a consequence, the macroscopic unraveling of the stalk as well as the slipping of the stalk or of the cardboard holders between the clamps become actually impossible due to the cooperative action of the high closure pressure and of the double-sided tape, so just the right stretching of the bundle itself becomes allowed. Moreover, no additional length is available for sliding after the clamps so we can exclude artifacts in our observations.

The diameter of the single thread is 6.03 µm (**Figure 4 d**), which is close to the upper value of the range from 1 to 6 µm indicated in [8], while the diameter of the bundles fell in the range from 200 to 300 µm. The FIB images gave us the opportunity to observe how many silk threads composed each stalk, thus allowing us to calculate the real thread cross-sectional area. Using the FESEM we saw that each stalkwas made up of an average of 150 single silk threads, corresponding to an effective cross section of 4283.67 µm^2^.

**Figure 5:**
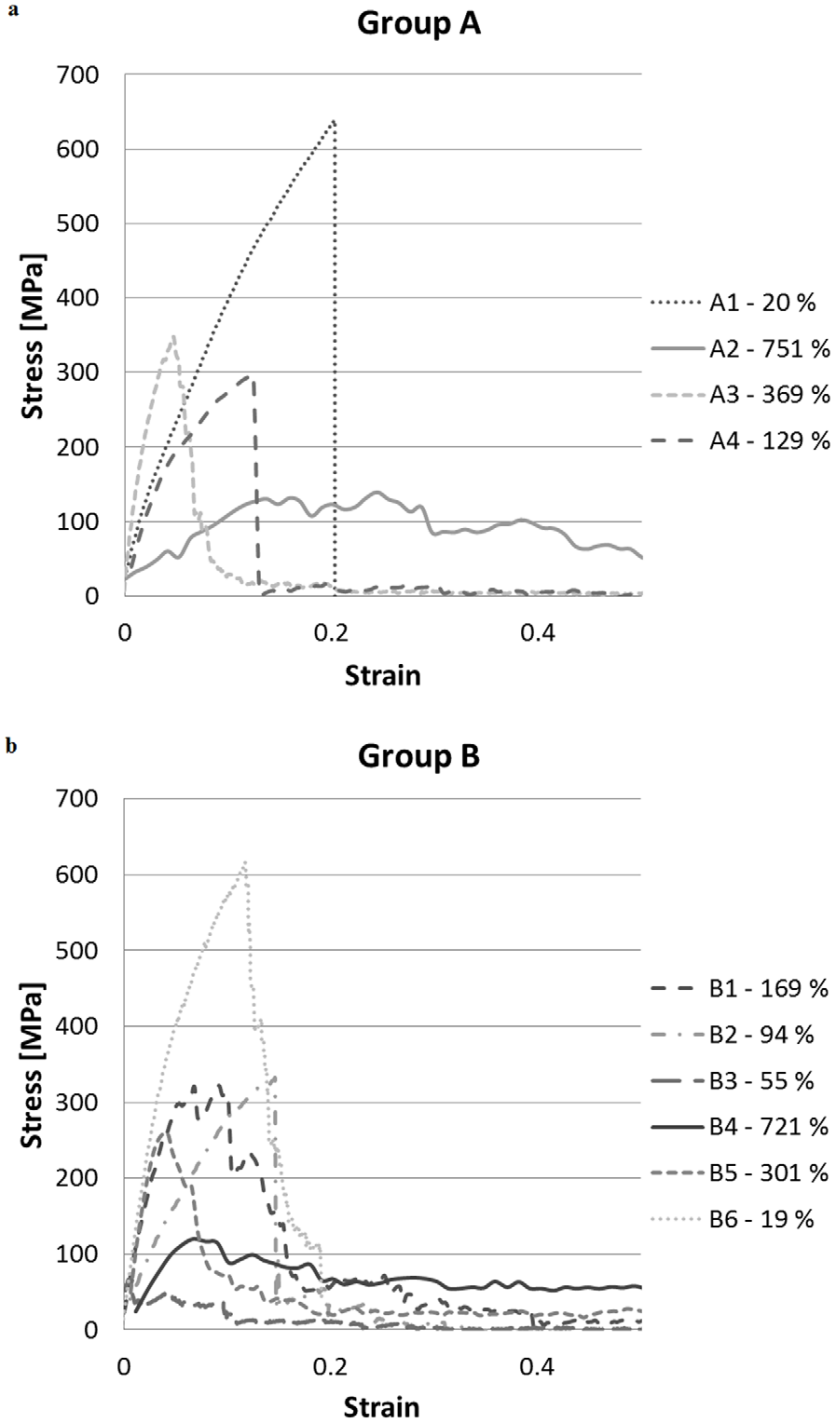
Stress - strain curves of group A (a) or B (b) stalks.

From the various tensile tests, we calculated the average failure stress, which for group A was 0.355 GPa and for group B was 0.286 GPa, even if very scattered. The average failure strain was 318% for group A and 227% for group B. The average values of toughness were 76.5 MJ/m^3^ for group A and 51.3 MJ/m^3^ for group B. Young's modulus is calculated as the initial slope of the stress-strain curve and equal to 20.4 GPa for group A and 22.46 GPa for group B. **Figure 5 a, b** shows the various stress-strain curves that were characterized.

The stress-strain curves showed different shapes, also caused by the varying number of threads that composed each stalk. The curves had a small initial elastic region which reached a maximum stress which then dropped quickly to very low values, but continuing to large strains until the failure was reached, in some cases through a series of peaks which were caused by the breaking of single or a small number of threads in the stalk. The strain values also differed, but were all above 20%, with some stalks reaching 300% strain or more before breaking. Two tests were pulled to an extraordinary length, the maximum strain that they were subject to was 751% for stalk A2, corresponding to a toughness value of 130.7 MJ/m^3^ (represented with solid line in **Figure 5 a**), and 721% for stalk B4, corresponding to a toughness value of 117.4 MJ/m^3^ (represented with solid line in **Figure 5 b**).

**Figure 6:**
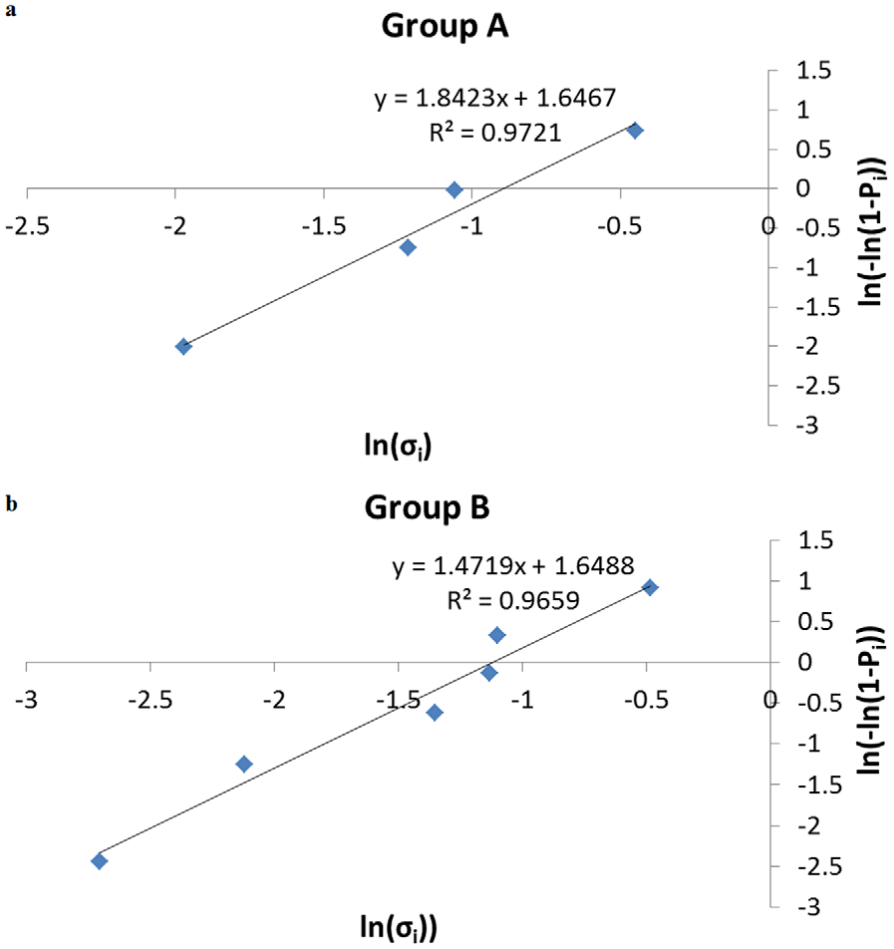
Weibull statistics for stress of group A (a) or B (b) stalks.

Following the Weibull statistics, we apply Eq. (4) to the set of fracture stresses of the egg sac silk stalks of *Meta menardi*, reported in **Table 2**. The Weibull modulus *m*, an index of the dispersion of the stress distribution, is 1.8 for group A (**Figure 6 a**) and 1.5 for group B (**Figure 6 b**), whereas *σ*_0_, an index of the mean value of the stress distribution, is equal to 0.409 GPa for group A and 0.326 GPa for group B. Note that the correlation coefficient is high (R^2^ = 0.97) for both the groups.

**Table 2:** The measured stress of each stalk, in ascending order.

Group A		Group B	
Test n°	Stress (GPa)	Test n°	Stress (GPa)
1 (A2)	0.139	1 (B3)	0.067
2 (A3)	0.297	2 (B4)	0.120
3 (A4)	0.347	3 (B5)	0.259
4 (A1)	0.639	4 (B1)	0.322
		5 (B2)	0.332
		6 (B6)	0.617

## Discussion

Referring to previous scientific studies, scientists focused their attention on different types of silk and mechanically characterized them. Limiting our analysis to tensile tests conducted on egg sac silk, only few studies have been carried out, particularly on *Argiope argentata* [3], *Araneus diadematus* [24], *Nephila madagascariensis* [29], *Argiope bruennichi* [33], *Araneus gemmoides* and *Nephila clavipes* [34]. In addition, the genuses *Nephila, Araneus* and *Meta* belong to three related families of orb web weavers (Nephilidae, Araneidae and Tetragnathidae, respectively [16]) and thus general conclusions could be drawn [49].The shapes of the stress-strain curves that we observed have a similar shape of that for carbon nanotube (CNT) bundles [50], [51]. These curves present a series of kinks or load drops which are an indication of sub-bundle failures when a bundle is pulled in a direction parallel to its axis. As we can see in our data, we also have a series of kinks indicating that the failure of the bundle, once it has reached its peak load, occurs with the fracture of sub-bundles. Though our curves were similar to those of CNT bundles, they were completely different to those of the dragline silk bundles and egg sac silk stalks [34]. Comparing their results to ours, we see that their failure stresses and toughness are much higher.

**Figure 7:**
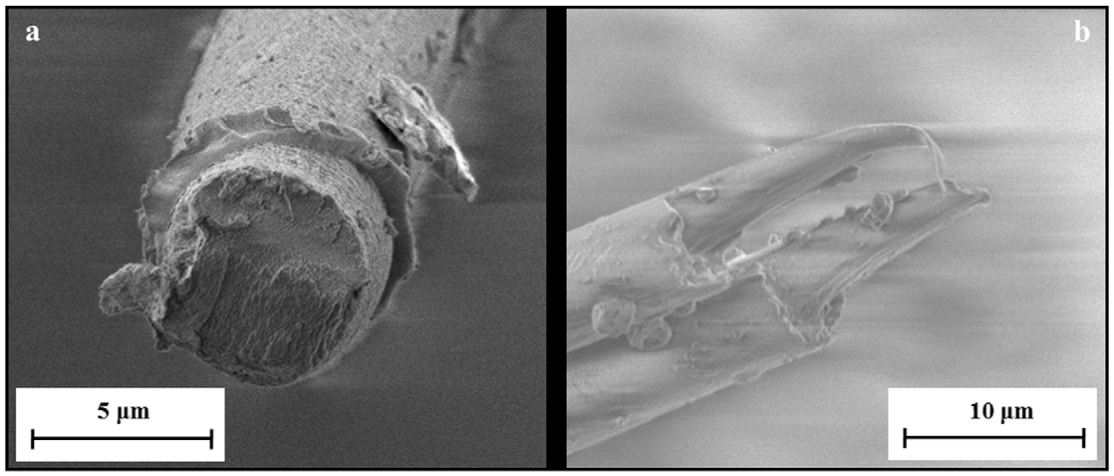
Detailed views of fracture surfaces of broken silk fibers.

The β-sheet nanocrystals are held together by hydrogen bonds, one of the weakest chemical bond. It was seen that when a thread is pulled, the force peaks in the force-displacement graph are a confirmation that the hydrogen bonds break and reform at an adjacent hydrogen bond ring. This occurs by preserving the initial side-chain orientation and shifting, or by rotating and forming an opposite side-chain orientation. This leads to a series of force peaks in the mechanical response and increases the total dissipated energy [2]. The size of the β-sheet nanocrystals influences the tensile response of a silk thread, consequently the smaller the crystals the greater the strength and toughness of the thread. As mentioned above, the fibers are made up of semi-amorphous α-chains and β-pleated sheets which are embedded in a rubber like matrix. Images from the FESEM further show that the fibers are made up of 2 layers [38], an inner layer and an outer coating. It seems that some fibers have a polymeric like fracture surface and some have a more regular surface. This second case is probably due to the different crystals that make up the fiber, in fact β-sheets are crystal-like, responsible for the toughness of the thread and have a more fragile rupture. On the other hand we can assume that some fibers have a very ductile break, caused by the amorphous rubber-like region (**Figure 7 a, b**).

**Figure 8:**
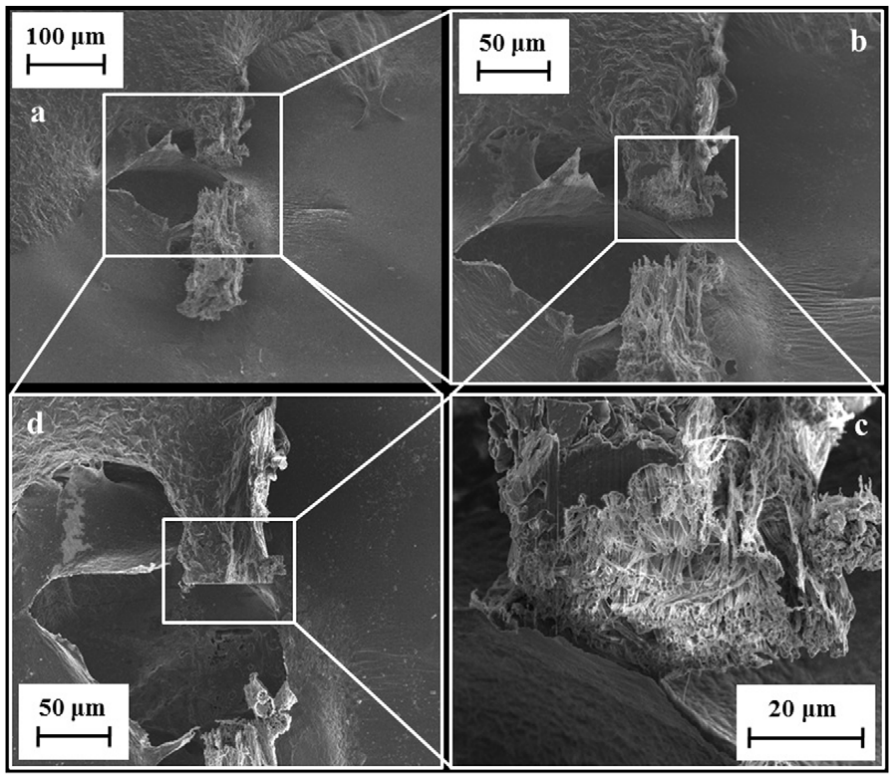
FESEM characterization of the stalk cut with FIB: (a, b, c) at an eye angle of 52°, (d) from the top.

Having cut our stalk with a FIB, we have been able to observe the cross section of our stalks at a SEM eye angle of 52° (**Figure 8 a, b, c**) and from the top (**Figure 8 d**) and thus calculate the stress-strain curves. Each stalkis made up of a series of single silk threads which, when pulled, stack up together to form what we initially hypothesized being a cylindrical cable. The diameters of our egg sac silk threads (∼6 µm) were slightly smaller than those of egg sac silk of *Nephila clavipes* (∼7 µm) [34] while equal to those of *Argiope bruennichi* [33], but much bigger than the dragline silk (∼1.4 µm) of the same species. For comparison, the diameters of dragline silk and minor ampullate in *Nephila clavipes* or *Araneus gemmoides*, were estimated to be 3 and 2.5 µm [34], or 2.5 and 2 µm [33], respectively.

Surprisingly, the strains that our fibers sustained were impressively high, some stalks were pulled to more than 200%, reaching values of 721–751%, which have not been seen in any spider single thread or stalk of egg sac silk yet. Such enormous elongations suggest a huge unrolling mechanism in the stalk.

**Figure 9:**
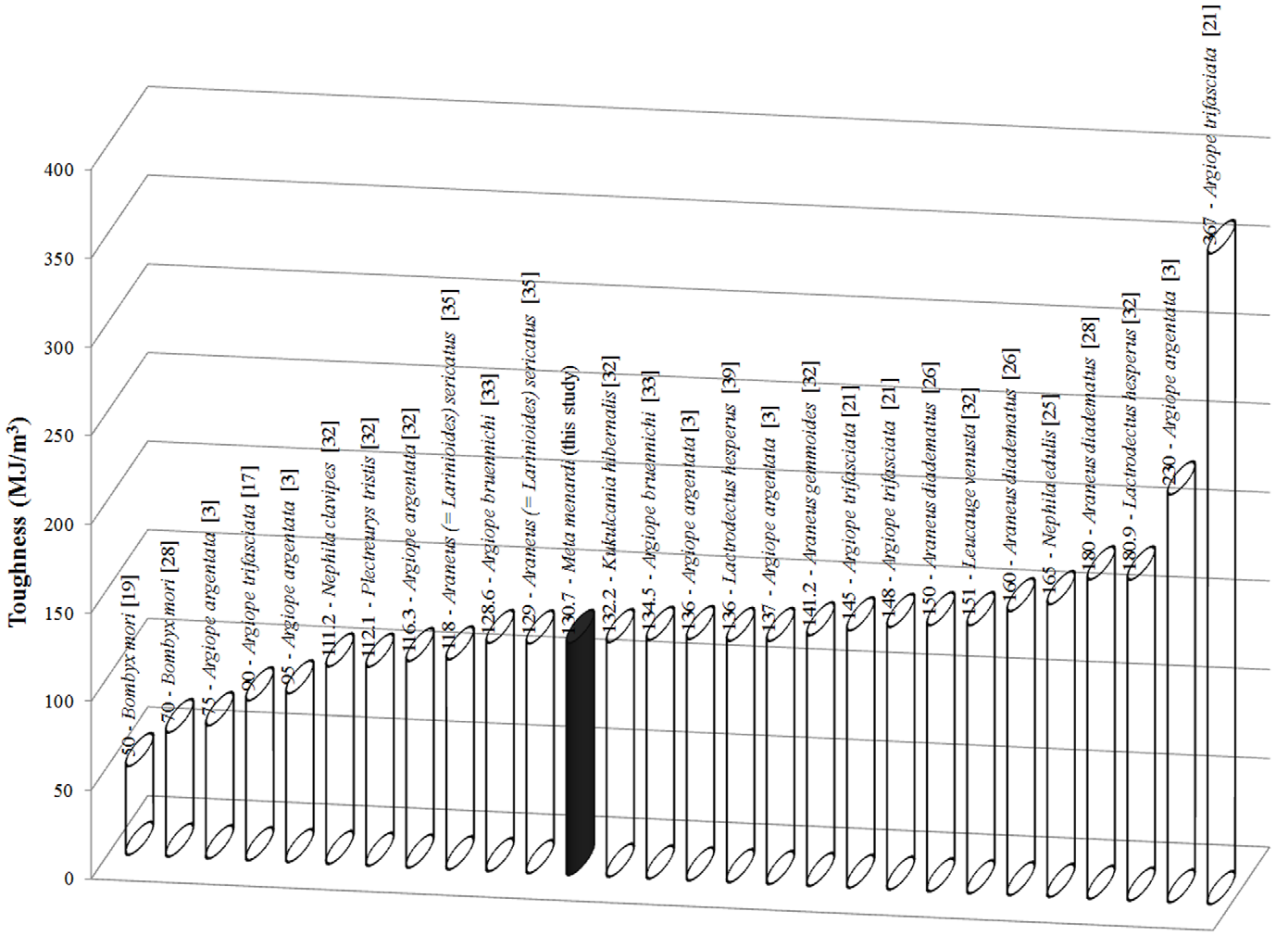
The maximum toughness of different types of (mainly spider) silks.

In **Figures 9** **,** **10** **,** **11**, we report toughness, ultimate stress and ultimate strain respectively for different types of spider silks; specifically in **Figure 11** our record of ultimate strain clearly emerges. The reason for this very high strain is yet unknown but could be caused by an interaction and different disposition of the α-chains and β-pleated sheets within the fibers thus giving them the possibility to stretch to such high strain values. As stated in the introduction, it has been observed that physical interactions between the fibers could influence the elongation data and so increased the stretching capabilities of the stalk, compared to that of the single fiber [34]. We saw that the extreme strain of the stalkscould be caused by a macroscopic unraveling of the stalkitself. The failure strains of the egg sac silk of *Araneus diadematus* reached values of 30–40%, much lower than our strains [24]. Egg sac threads from *Nephila clavipes* extended 24±2% their initial length and the maximum stress was 1.3±0.2 GPa whereas for *Araneus gemmoides* these values were respectively 19±2% and 2.3±0.2 GPa [34].

**Figure 10:**
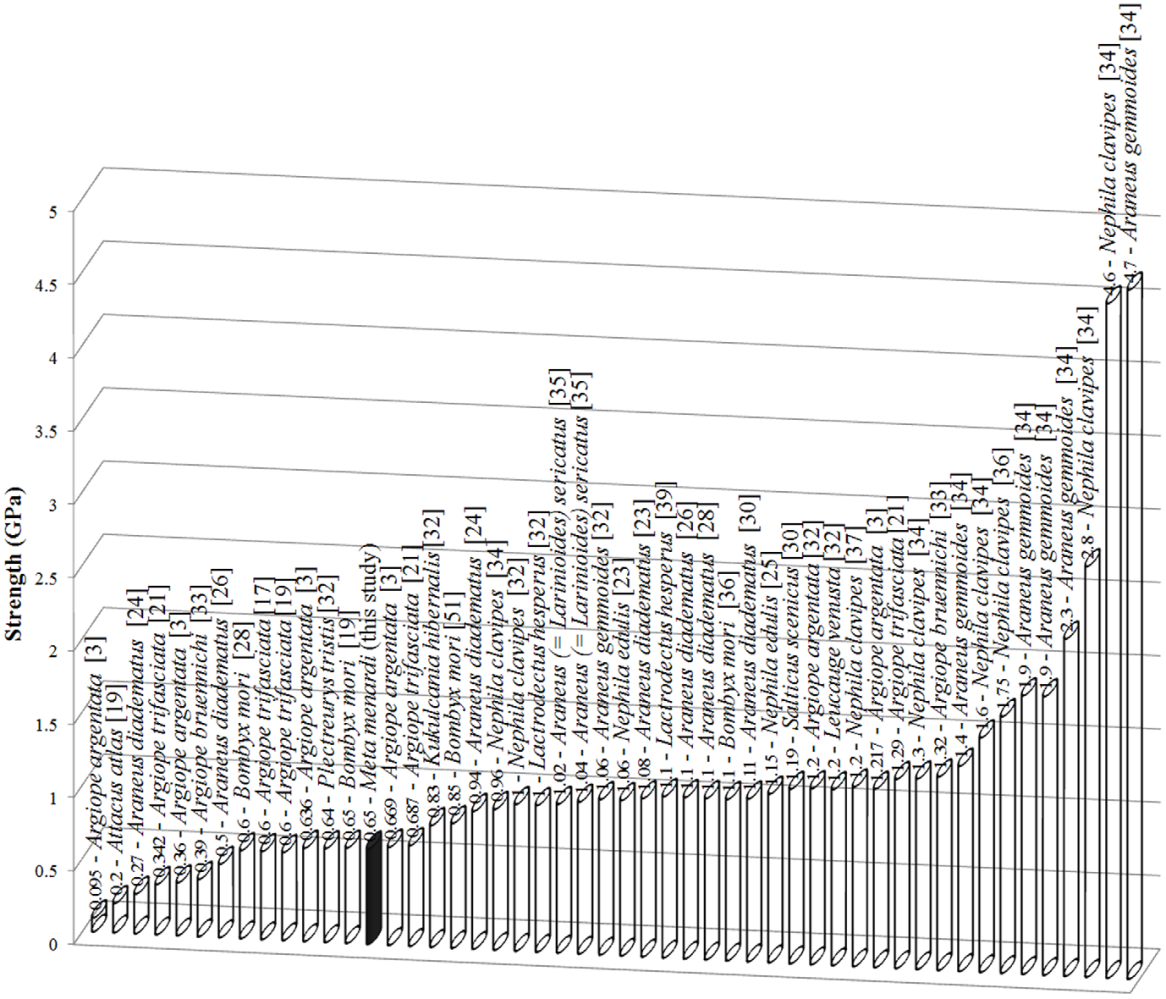
The maximum strength of different types of (mainly spider) silks.

**Figure 11:**
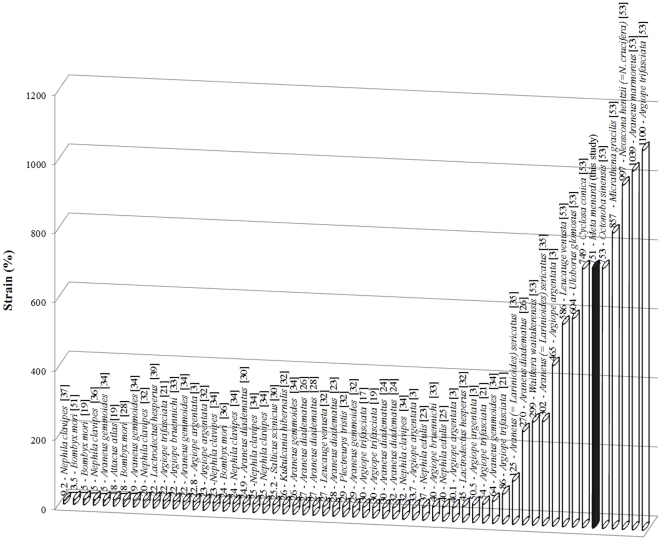
The maximum strain of different types of (mainly spider) silks, showing the record for egg sac silk stalks observed in our experiments.

The failure stresses of our stalks were much inferior to these, but the strains sustained by them were much higher, probably due to physical interactions within the stalks and the type of deformation that occurred at the nanoscale. Bundles of dragline and minor ampullate silk made up of 100 threads were also tested [34] and showed a wide range over which they broke and thus these authors were not able to determine a useful value for the tensile strength of the fiber bundles due to the fact that there was a great variety in the diameters of the threads that made up the bundle [34]. We have here solved this problem using Weibull statistics and our results (*m* is in the range from 1.5 to 1.8 and *σ*_0_ is in the range from 0.33 to 0.41 GPa) are in line with the values of the shape (*m*) and scale (*σ*_0_) parameters of Weibull statistics which are equal to 3.4 and 0.6 GPa for the dragline of *Argiope trifasciata* [17] or equal to 5.7 and 0.4 GPa for the silkworm cocoons of *Bombyx mori* [52], respectively.

The great standard deviation in the values of our stress and strain results within the two groups of stalks could be due to the fact that they differed in terms of diameter, number of threads and the physical condition of the stalks that also may affect the performances, see **Table S1**. The stalks were all taken from the natural habitat of the spider where humidity and temperature play an important role. As it was seen moisture induces supercontraction in the threads thus causing them to tighten up [20], the temperature of the caves was roughly 9±2°C, while the tests were done in an environment where the temperature was much higher and could have caused the fibers to change their natural state. The tests were also done a couple of days after collecting the stalks and were kept in the laboratory in different conditions, causing the threads to lose or modify some properties.

We observed that the higher the stress that the stalkcould sustain, the lower the maximum strain before breakage. If strain reached high values the peak stresses did not exceed 0.64 GPa. In this case, we assume that the thread deformed in a rubber like way, extending to great values, due to physical interactions [53]–[57] between the threads composing the stalk.

### Conclusion

The tensile properties and the Weibull shape and scale parameters of stalks of egg sac silk of *Meta menardi*, obtained directly from their natural habitat, were determined here. The results that were gathered from the tests differed significantly when compared to other tensile tests on spider silk. Whether the comparison is done with egg sac silk from other species of orbweb weavers, dragline silk or minor ampullate silk, the results are much higher, up to 750%, to those reported in all the previous studies in terms of maximum strain of egg sac silk, suggesting the discovery of the most stretchable egg sac silk stalk ever tested. Such enormous elongations suggests a huge unrolling microscopic mechanism of the macroscopic stalk that, as a continuation of the protective egg sac, is expected to be composed by fibres very densely and randomly packed.

## Supporting Information

Table S1 **The main parameters which may influence tensile testing results: systematics, function, silk-producing glands, temperature and humidity, initial length (** ***l*** **_0_) of samples, number of tested threads, selected strain rate and number of tested samples.** Spider nomenclature according to [16].
(DOC)
