# Supplementary material for: Evidence of the Most Stretchable Egg Sac Silk Stalk, of the European Spider of the Year Meta menardi
Source: PLoS One. 2012 Feb 8;7(2):e30500. doi: 10.1371/journal.pone.0030500 (PMC3275603; doi:10.1371/journal.pone.0030500)
Supplement: Table S1 — The main parameters which may influence tensile testing results: systematics, function, silk-producing glands, temperature and humidity, initial length ( l 0) of samples, number of tested threads, selected strain rate and number of tested samples. Spider nomenclature according to [16]. (DOC) [file pone.0030500.s001.doc]

| **References** | **Class, Order** | **Family** | **Species** | **Function** | **Glands** | **Experimental conditions (temperature and humidity)** | **l_0_** | **Number of threads** | **Strain rate** | **Number of samples** |
| --- | --- | --- | --- | --- | --- | --- | --- | --- | --- | --- |
| [24] | Arachnida, Araneae | Araneidae | *Araneus diadematus* | egg sac | tubuliform (cylindrical) | wet (20 °C, 65 %) | 20 mm | 1 | 20 mm/min | 398 |
| [24] | Arachnida, Araneae | Araneidae | *Araneus diadematus* | structural threads and dragline | major ampullate | wet (20 °C, 65 %) | 20 mm | 1 | 20 mm/min | 183 |
| [26] | Arachnida, Araneae | Araneidae | *Araneus diadematus* | structural threads and dragline | major ampullate | not given | not given | not given | not given | not given |
| [28] | Arachnida, Araneae | Araneidae | *Araneus diadematus* | structural threads and dragline | major ampullate | not given | not given | not given | not given | not given |
| [23] | Arachnida, Araneae | Araneidae | *Araneus diadematus* | structural threads and dragline | major ampullate | wet (24 °C, 50 %) | 6.9 mm | 1 | 3 mm/min | 30 |
| [30] | Arachnida, Araneae | Araneidae | *Araneus diadematus* | structural threads and dragline | major ampullate | not given | not given | 2 | 14-20 mm/min | 16 |
| [26] | Arachnida, Araneae | Araneidae | *Araneus diadematus* | glue coating on viscid capture threads | aggregate glands | not given | not given | not given | not given | not given |
| [34] | Arachnida, Araneae | Araneidae | *Araneus gemmoides* | auxiliary  radial threads and temporary scaffolding | minor ampullate | not given | 40 mm | 1 | 5 mm/min | 10 |
| [34] | Arachnida, Araneae | Araneidae | *Araneus gemmoides* | egg sac | tubuliform (cylindrical) | not given | 40 mm | 1 | 5 mm/min | 10 |
| [34] | Arachnida, Araneae | Araneidae | *Araneus gemmoides* | structural threads and dragline | major ampullate | not given | 40 mm | 1 | 5 mm/min | 10 |
| [32] | Arachnida, Araneae | Araneidae | *Araneus gemmoides* | structural threads and dragline | major ampullate | not given | not given | 1 | 1 % strain/s | 23 |
| [34] | Arachnida, Araneae | Araneidae | *Araneus gemmoides* | auxiliary  radial threads and temporary scaffolding | minor ampullate | not given | 40 mm | 100 | 5 mm/min | 10 |
| [34] | Arachnida, Araneae | Araneidae | *Araneus gemmoides* | structural threads and dragline | major ampullate | not given | 40 mm | 100 | 5 mm/min | 10 |
| [35] | Arachnida, Araneae | Araneidae | *Araneus (= Larinioides) sericatus* | structural threads and dragline | major ampullate | wet (21 °C, 50 %) | 25 mm | 1 | 13.2 mm/min | 60 |
| [35] | Arachnida, Araneae | Araneidae | *Araneus (= Larinioides) sericatus* | glue coating on viscid capture threads | aggregate glands | wet (21 °C, 50 %) | 25 mm | 1 | 217.1 mm/min | 41 |
| [53] | Arachnida, Araneae | Araneidae | *Araneus marmoreus* | adhesive threads of the catching spiral | flagelliform glands | wet (23 °C, 60 %) | 20 mm | 1 | 0.017 mm/min | 3 |
| [3] | Arachnida, Araneae | Araneidae | *Argiope argentata* | structural threads and dragline | major ampullate | wet (21.5 °C, 45 %) | 21 mm | 1 | 12.6 mm/min (1 % strain/s) | 13 |
| [32] | Arachnida, Araneae | Araneidae | *Argiope argentata* | structural threads and dragline | major ampullate | not given | not given | 1 | 1 % strain/s | 62 |
| [3] | Arachnida, Araneae | Araneidae | *Argiope argentata* | wrapping silk and packing silk | aciniform gland | wet (21.5 °C, 45 %) | 10 mm | 1 | 6 mm/min (1 % strain/s) | 28 |
| [3] | Arachnida, Araneae | Araneidae | *Argiope argentata* | adhesive capture threads of the catching spiral | flagelliform glands | wet (21.5 °C, 45 %) | 21 mm | 1 | 12.6 mm/min (1 % strain/s) | 87 |
| [3] | Arachnida, Araneae | Araneidae | *Argiope argentata* | auxiliary  radial threads and temporary scaffolding | minor ampullate | wet (21.5 °C, 45 %) | 21 mm | 1 | 12.6 mm/min (1 % strain/s) | 51 |
| [3] | Arachnida, Araneae | Araneidae | *Argiope argentata* | egg sac | tubuliform (cylindrical) | wet (21.5 °C, 45 %) | 21 mm | 1 | 12.6 mm/min (1 % strain/s) | 29 |
| [33] | Arachnida, Araneae | Araneidae | *Argiope bruennichi* | egg sac | tubuliform (cylindrical) | wet (24 °C, 34 %) | 20 mm | 1 | 10 mm/min | 4 |
| [33] | Arachnida, Araneae | Araneidae | *Argiope bruennichi* | structural threads and dragline | major ampullate | wet (24 °C, 34 %) | 20 mm | 1 | 10 mm/min | 4 |
| [17] | Arachnida, Araneae | Araneidae | *Argiope trifasciata* | structural threads and dragline | major ampullate | wet (20 °C, 60 %) | 20 mm | 1 | 0.24 mm/min (2*10^-4^ /s) | 28 |
| [21] | Arachnida, Araneae | Araneidae | *Argiope trifasciata* | structural threads and dragline | major ampullate | not given | 21 mm | 1 | 12.6 mm/min (1 % strain/s) | 7 |
| [19] | Arachnida, Araneae | Araneidae | *Argiope trifasciata* | structural threads and dragline | major ampullate | wet (20 °C, 60 %) | 10 mm | 1 | 0.12 mm/min (2*10^-4^ /s) | 10 |
| [21] | Arachnida, Araneae | Araneidae | *Argiope trifasciata* | wrapping silk and packing silk | aciniform gland | not given | 10 mm | 2 | 6 mm/min (1 % strain/s) | 14 |
| [21] | Arachnida, Araneae | Araneidae | *Argiope trifasciata* | auxiliary  radial threads and temporary scaffolding | minor ampullate | not given | 21 mm | 2 | 12.6 mm/min (1 % strain/s) | 11 |
| [53] | Arachnida, Araneae | Araneidae | *Argiope trifasciata* | adhesive threads of the catching spiral | flagelliform glands | wet (23 °C, 60 %) | 20 mm | 1 | 0.017 mm/min | 3 |
| [53] | Arachnida, Araneae | Araneidae | *Micrathena gracilis* | adhesive threads of the catching spiral | flagelliform glands | wet (24 °C, 60 %) | 20 mm | 1 | 0.017 mm/min | 3 |
| [53] | Arachnida, Araneae | Araneidae | *Neoscona hentzii (= N. crucifera)* | adhesive threads of the catching spiral | flagelliform glands | wet (23 °C, 60 %) | 20 mm | 1 | 0.017 mm/min | 3 |
| [53] | Arachnida, Araneae | Araneidae | *Cyclosa conica* | adhesive threads of the catching spiral | flagelliform glands | wet (25 °C, 61 %) | 20 mm | 1 | 0.017 mm/min | 3 |
| [53] | Arachnida, Araneae | Uloboridae | *Octonoba sinensis* | dry cribellar capture threads of the catching spiral | flagelliform glands | wet (23 °C, 61 %) | 20 mm | 1 | 0.017 mm/min | 3 |
| [53] | Arachnida, Araneae | Uloboridae | *Uloborus glomosus* | dry cribellar capture threads of the catching spiral | flagelliform glands | wet (24 °C, 62 %) | 20 mm | 1 | 0.017 mm/min | 3 |
| [53] | Arachnida, Araneae | Uloboridae | *Waitkera waitakerensis* | dry cribellar capture threads of the catching spiral | flagelliform glands | wet (25 °C, 70 %) | 20 mm | 1 | 0.017 mm/min | 3 |
| [32] | Arachnida, Araneae | Filistatidae | *Kukulcania hibernalis* | dragline | major ampullate | not given | not given | 1 | 1 % strain/s | 102 |
| [32] | Arachnida, Araneae | Theridiidae | *Lactrodectus hesperus* | structural threads and dragline | major ampullate | not given | not given | 1 | 1 % strain/s | 70 |
| [39] | Arachnida, Araneae | Theridiidae | *Lactrodectus hesperus* | auxiliary  radial threads and temporary scaffolding | minor ampullate | not given | 12 mm | 30 | 12.6 mm/min | 30 |
| [32] | Arachnida, Araneae | Tetragnathidae | *Leucauge venusta* | structural threads and dragline | major ampullate | not given | not given | 1 | 1 % strain/s | 61 |
| [53] | Arachnida, Araneae | Tetragnathidae | *Leucauge venusta* | adhesive threads of the catching spiral | flagelliform glands | wet (25 °C, 60 %) | 20 mm | 1 | 0.017 mm/min | 3 |
| this study | Arachnida, Araneae | Tetragnathidae | *Meta menardi* | egg sac | tubuliform (cylindrical) | wet (22 °C, 31 %) | 18-19 mm | 150 | 2 mm/min | 10 |
| [34] | Arachnida, Araneae | Nephiliidae | *Nephila clavipes* | auxiliary  radial threads and temporary scaffolding | minor ampullate | not given | 40 mm | 1 | 5 mm/min | 10 |
| [34] | Arachnida, Araneae | Nephiliidae | *Nephila clavipes* | egg sac | tubuliform (cylindrical) | not given | 40 mm | 1 | 5 mm/min | 10 |
| [34] | Arachnida, Araneae | Nephiliidae | *Nephila clavipes* | structural threads and dragline | major ampullate | not given | 40 mm | 1 | 5 mm/min | 10 |
| [32] | Arachnida, Araneae | Nephiliidae | *Nephila clavipes* | structural threads and dragline | major ampullate | not given | not given | 1 | 1 % strain/s | 66 |
| [36] | Arachnida, Araneae | Nephiliidae | *Nephila clavipes* | structural threads and dragline | major ampullate | wet (23 °C, 49 %) | 12.7 mm | 1 | 12.7 mm/min (100 %/min) | 19 |
| [37] | Arachnida, Araneae | Nephiliidae | *Nephila clavipes* | structural threads and dragline | major ampullate | wet (21 °C, 50 %) | 50.8 mm | 1 | 304.8 mm/min (10 % strain/s) | 30 |
| [34] | Arachnida, Araneae | Nephiliidae | *Nephila clavipes* | auxiliary  radial threads and temporary scaffolding | minor ampullate | not given | 40 mm | 100 | 5 mm/min | 10 |
| [34] | Arachnida, Araneae | Nephiliidae | *Nephila clavipes* | structural threads and dragline | major ampullate | not given | 40 mm | 100 | 5 mm/min | 10 |
| [23] | Arachnida, Araneae | Nephiliidae | *Nephila edulis* | structural threads and dragline | major ampullate | wet (24 °C, 50 %) | 6.9 mm | 1 | 3 mm/min | 30 |
| [25] | Arachnida, Araneae | Nephiliidae | *Nephila edulis* | structural threads and dragline | major ampullate | wet (22 °C, 50 %) | 12 mm | 1 | 6 mm/min (50 % strain/min) | not given |
| [32] | Arachnida, Araneae | Plectreuridae | *Plectreurys tristis* | dragline | major ampullate | not given | not given | 1 | 1 % strain/s | 108 |
| [30] | Arachnida, Araneae | Salticidae | *Salticus scenicus* | dragline | major ampullate | not given | not given | 2 | 14-20 mm/min | 5 |
| [19] | Insecta, Lepidoptera | Saturniidae | *Attacus atlas* | cocoon | silk glands | wet (20 °C, 60 %) | 30 mm | 1 | 0.36 mm/min (2*10^-4^ /s) | 10 |
| [28] | Insecta, Lepidoptera | Bombycidae | *Bombyx mori* | cocoon | silk glands | not given | not given | not given | not given | not given |
| [19] | Insecta, Lepidoptera | Bombycidae | *Bombyx mori* | cocoon | silk glands | wet (20 °C, 60 %) | 30 mm | 1 | 0.36 mm/min (2*10^-4^ /s) | 10 |
| [51] | Insecta, Lepidoptera | Bombycidae | *Bombyx mori* | cocoon | silk glands | wet (20 °C, 60 %) | 40 mm | 1 | 0.48 mm/min (0.0002 /s) | 10 |
| [36] | Insecta, Lepidoptera | Bombycidae | *Bombyx mori* | cocoon | silk glands | wet (23 °C, 49 %) | 12.7 mm | 1 | 3.81 mm/min (30 %/min) | 20 |
